# Proactive Personality and Social Support With Pre-retirement Anxiety: Mediating Role of Subjective Career Success

**DOI:** 10.3389/fpsyg.2021.569065

**Published:** 2021-07-02

**Authors:** Lawrence Ejike Ugwu, Ibeawuchi K. Enwereuzor, Barnabas E. Nwankwo, Stella Ugwueze, Franscisca N. Ogba, Evelyn E. Nnadozie, Chinyere O. Elom, Angela Eze, Michael A. Ezeh

**Affiliations:** ^1^Psychology Department, Renaissance University Ugbawka, Enugu, Nigeria; ^2^Psychology Department, University of Nigeria, Nsukka, Nigeria; ^3^Department of Psychology, Caritas University, Enugu, Nigeria; ^4^Educational Foundation, Alex Ekwueme Federal University Ndufu-Alike Ikwo, Ebonyi, Nigeria; ^5^Art and Humanities Education, Alex Ekwueme Federal University Ndufu-Alike Ikwo, Ebonyi, Nigeria; ^6^Psychology Department, Enugu State University of Science and Technology, Enugu, Nigeria

**Keywords:** subjective career success, social support, pre-retirement anxiety, financial preparedness, social obligation, social alienation

## Abstract

The main objective of this paper is to investigate the mediating role of subjective career success (SCS) in the relationship between proactive personality, social support (SS), and pre-retirement anxiety. Using a two-wave longitudinal design, 624 pre-retirees were sampled (*M* = 56.49 years; *SD* = 4.56); of these, 237 (37.98%) were males and 387 (62.02%) were females. Measurement model and mediation test were performed using the SmartPLS and IBM SPSS Amos software. The result indicated that proactive personality, SS, and SCS showed negative relationships with the dimensions of pre-retirement anxiety (financial preparedness, social obligation, and social alienation). Subjective career success mediated the relationship between proactive personality and pre-retirement anxiety.

## Introduction

The anxiety that comes with retirement could be as a result of perceived unpreparedness and lack of self-adequacy or perceived devastation and traumatic experiences in the life of some retirees for whom the approaching retirement is unknown. The adverse psychological and socioeconomic disposition identified to characterize retired civil servants due to functional discontinuation of their regular financial source of livelihood and its corresponding decline in social status cannot be overstated (Yeung and Zhou, 2017). Such challenges include financial insufficiency, declining health, dysfunctional family matters, and psychological or behavioral disorders, such as depression, hypertension, identity crisis, loneliness, and fast aging occasioned sometimes by a lack of good accommodation among others (Tokunboh, 1998). Some also experience loss of self-esteem due to lower status in which they imagine themselves on retirement from a highly exalted position they had occupied as public servants (Hansson et al., 2019). Therefore, there is a need to investigate the perception and attitude of employees toward pre-retirement anxiety.

Several studies have explored retirement, but very few studies have explained the role of pre-retirement or how personality variables like proactiveness and social support (SS) influence pre-retirement anxiety. This study is filling the gap of exploring pre-retirement anxiety. It is also examining the role proactive personality plays in alleviating the anxiety at pre-retirement as proactive personality has been established to boost satisfaction at work and retirement (e.g., Jawahar and Liu, 2016; Kuo et al., 2019). Moreover, it is also concerned with the roles of various dimensions of SS (family, friends, and significant others) in helping potential retirees adjust to the impending retirement. According to Kubicek et al. (2010), some resources at work and personal life of an individual can have an indirect effect on retirement to determine the quality of life. The current study presumes that career success could mediate the relationship between proactive personality, social support, and pre-retirement anxiety.

The focus on pre-retirement anxiety is pertinent because of the nonexistence of proper research on it in Africa and especially in Nigeria. Workers are not aware of the retirement scheme used in their organizations, this resulted in fewer workers (8.1% of the working population) participating in the contributory pension scheme was introduced in 2004 (Pricewaterhousecooper, 2016). There is a high distrust in government as most of the pension savings are embezzled by some of its administrators (Abu and Musari, 2012; Nweke, 2014). The study of pre-retirement is justified because retirement challenges have precursors; these precursors if well taken care of, could most likely prevent some future retirement challenges. Proactive personality, social support, and career success are possible factors that can alleviate pre-retirement anxiety (Li et al., 2010; Maurer and Chapman, 2017; Kuo et al., 2019). Moreover, Kubicek et al. (2010) posited that some resources at work and personal life of an individual can have an indirect effect on retirement to determine the quality of life. The current study presumes that career success could mediate the relationship between proactive personality, social support, and pre-retirement anxiety. These assumptions could go a long way in discovering the pattern of relationship that exists between these variables and of course fill the gap in the literature. There is rarely any study to our knowledge that has considered the role proactive personality and social support play in pre-retirement anxiety. This study improves on the growing literature on retirement (Wang et al., 2016; Zhu and Walker, 2018) and the need to understand how developing economies manage to cope during this transition period. Retirement plans for workers in these economies are not admirable, but retirees have no choice than to survive. This study would go a long way in providing insights on how to get ready before the inevitable retirement.

### Proactive Personality and Pre-Retirement Anxiety

Proactive personality is a relatively stable tendency characterized by forecasting future changes, planning, and perseverance (Bateman and Crant, 1993). It also denotes the dispositional tendency to engage in proactive behavior in a variety of situations. Individuals with a proactive personality are inclined to change their circumstances intentionally, including their physical environment (McCormick et al., 2018).

Workers who possess the proactive personality trait tend to strive for a specific goal. They make things happen and are generally good and maneuvering situations toward their desired outcome. They identify opportunities, take action, and persevere until they bring about meaningful change (Crant, 2000). Although some people react to, adapt to, and are shaped by their environments, proactive people take personal initiative to have an impact on the world around them.

While several studies have investigated the relationship between proactive personality and variables, such as age, job success, job performance, organizational productivity, entrepreneurial success, and organizational citizenship behavior (Gan and Cheung, 2010; Feldman and Beehr, 2011; Bakker et al., 2012), to the best of the knowledge of authors, no study focused explicitly on the relationship between proactive personality and pre-retirement or retirement anxiety. However, we can draw inferences from existing studies to develop our hypotheses.

For example, Feldman and Beehr (2011) observed that individuals who are highly conscientious may view any drop in performance as a sign of poor fit, whereas individuals who are highly agreeable may be more attuned to positive social feedback than to negative task feedback in their jobs. Conscientiousness is the personality trait of being careful or diligent and implies a desire to do a task well and to take obligations to others seriously (Major et al., 2006). It is important to note that workers who have high proactive personality traits are highly conscientious. Conscientious workers tend to be efficient and organized as opposed to easy-going and disorderly. Other studies (Sahin, 2006; Ozkurt and Alpay, 2018) noted the role proactive personality plays in converting anxiety troubles and mistakes of life to new experiences.

Robinson et al. (2010) found that neuroticism was related to a negative view of circumstances leading to retirement, whereas conscientiousness was related to aspirational reasons for retirement. Filer and Petri (1988) posited that openness and social skills are associated with later retirement, net of cognitive and physical demands, and other job characteristics. One of the goals of this study is to directly study proactive personality and pre-retirement anxiety.

In the event of loss of work roles, social ties facilitate easy adjustment (van Solinge and Henkens, 2008; Dulin et al., 2011). However, a longitudinal study by Reitzes and Mutran (2006) observed that pre-retirement unique relationships and social support characteristics influence initial retirement adjustment and later life. We therefore hypothesize:

*H1: Proactive personality will negatively predict pre-retirement anxiety [(a) financial preparedness, (b) social obligation, and (c) social alienation].*

#### Social Support and Pre-retirement Anxiety

Social support is the perception and actuality that one is cared for, has assistance available from other people, and that one is part of a supportive social network (Taylor, 2011). Extended family, friends, church members, neighbors, and fictive kin constitute complementary sources of support (Billingsley, 1992; Dilworth-Anderson et al., 1993; Chatters et al., 1994; Taylor et al., 1997). In Africa, our collectivistic culture: community lifestyle and extended family system make social support imperative (Centre for Social Justice, 2010; Okasha et al., 2012; Olaore and Drolet, 2017). These sources of support may assume greater prominence under conditions of social, emotional, economic, and demographic changes like approaching retirement. These supports can be emotional (e.g., nurturance), tangible (financial assistance), informational (advice), or companionship (sense of belonging). The lack of a social support system and intimate social contacts are more likely to induce loneliness, depression, and anxiety for older adults (Green et al., 2001). Such close social contacts are important sources of social support in later life. Social support from an interpersonal communication viewpoint is understood as supportive behavior performed for an individual by others and is often assessed by the perception of received support from an individual (Burleson and MacGeorge, 2002; Goldsmith, 2004). Thus, the current study considers its implication in the pre-retirement anxiety of civil servants in Nigeria.

Contact with friends and family has been associated with better health (Lee and Ishii-Kuntz, 1987; Thompson and Heller, 1990), and satisfaction with support has been shown to have a positive effect on well-being (Chi and Chou, 2001). A greater number of individuals approaching retirement will be reaching old age. It is imperative that certainties and social demands of life on older adults are investigated and well understood so that reliable information can be used in promoting the care, health, and independence of older adults. Life events can have a significant effect on social support with older adults who are more susceptible to changes as a result of age-specific events, such as retirement (Gurung et al., 2003).

Retired individuals have been shown to experience increased contact with friends outside of work, possibly to compensate for the loss of work-related social ties (Fox, 1977). Contact with family also usually increases among retirees due to greater available time (Price, 1992; van Tilburg, 1992). This new-found free time showed that grandfathers like family moments with children and grandchildren than going out, while grandmothers like it, they connect more with beyond-family contacts (Field and Minkler, 1988; Sapadin, 1988; Stacey-Konnert and Pynoos, 1992; Myers and Booth, 1996).

Quality friendships have been shown to lower social anxiety and depression among retirees and enhance the quality of life (Krabill and Aday, 2005; Rodebaugh et al., 2015). Potts (1997) in his study found that the association with friends far away from the community of an individual plays a better role in reducing anxiety and depression than close community relationships. However, Kasper et al. (2019) in their exploratory study in four countries (United States, the Netherlands, Thailand, and Malaysia) showed that the relationship with friends had no significant effect on the financial satisfaction, except in the United States, which showed a negative relationship. Harris (2013) clearly stated that friendship is an essential ingredient in retirees as they grow older and weary in physical health and mind, the friends they kept over the years are the people who would still be around in the worse times of their lives. We therefore hypothesize:

*H2: Social support will negatively predict pre-retirement anxiety [(a) financial preparedness, (b) social obligation, and (c) social alienation].*

#### Subjective Career Success and Pre-retirement Anxiety

Subjective career success (SCS) refers to the enjoyment of work, pride in accomplishments, satisfaction in their work, and connection with colleagues of employees (Groysberg and Abrahams, 2014). The paucity of studies in SCS narrowed the satisfaction of workers to mostly objective career success, which included financial benefits, promotions, and much more tangible rewards. Few researchers like van Dam et al. (2009) inferred that workers high in subjective career success are more likely to postpone retirement by taking up other similar jobs than those with low subjective career success. Its role with pre-retirement anxiety has not been studied. It is important to note that at this stage of pre-retirement, the issues in developmental theory of Erikson (1982), the concept of integrity vs. despair comes with some concerns of ego integrity, depression, and anxiety. Those with high subjective career success could experience higher levels of integrity than despair. They have little to regret and much to be happy about. This is supported by some studies that have found a negative relationship between ego integrity and anxiety (James and Zarrett, 2005; Phillips and Ferguson, 2013). One of the rationales of this study was to find out if subjective career success, which depicts satisfaction with one’s career, might predict pre-retirement anxiety.

*H3: Subjective career success will negatively predict pre-retirement anxiety [(a) financial preparedness, (b) social obligation, and (c) social alienation].*

#### Subjective Career Success as a Mediator

Career success refers to the extent and ways in which an individual can be described as successful in his/her work–life so far (Gunz and Heslin, 2005; Ng and Feldman, 2014). According to Seibert et al. (1999), career success can be viewed as objective and subjective. Objective career success explains the incremental high salary, job title, prestigious firm that one works in, awards, and accolades. While subjective career success is the enjoyment of work, pride in accomplishments, job satisfaction, and connection with colleagues (Groysberg and Abrahams, 2014). Researchers (Abele and Spurk, 2009; Dyke and Duxbury, 2010) have studied the significance of subjective career success along the post-modernization of career development. Success has transitioned from a traditional career development model to a no boundary/borderless career development approach. On the one hand, a few studies have examined subjective career success as a mediating variable (Erdogan et al., 2012; De Vos and Segers, 2013; Amah, 2014; Liu et al., 2015). On the other hand, De Coen et al. (2015) showed that there was a significant negative relationship between career satisfaction and retirement. However, Armstrong-Stassen and Cattaneo (2010) pointed that those with higher career success experienced lower retirement anxiety. By reviewing the literature, no study evaluated the mediating role of subjective career success in the relationship between proactive personality or social support and pre-retirement anxiety. Furthermore, as described previously, there is a direct relationship between subjective career success and proactive personality (Seibert et al., 1999), social support (Powell and Maineiro, 1992; Nabi, 2001), and related constructs to retirement anxiety (Calvo et al., 2007; Wang, 2007; Wang and Bodner, 2007; Horner, 2012; Luhmann et al., 2012). Thus, we assume that subjective career success may also mediate the relationship between proactive personality, social support, and pre-retirement anxiety, where an individual who is satisfied with his/her career as a result of having appropriate and sufficient proactive personality, in turn, may be more satisfied with his/her life and possess a high level of positive affect and a low level of negative affect. From the above discussion, we suppose a mediating role of subjective career success between both proactive personality and social support with pre-retirement anxiety.

Therefore, we hypothesize the following hypotheses:

*H4: Subjective career success will significantly mediate the relationship between proactive personality and pre-retirement anxiety [(a) financial preparedness, (b) social obligation, and (c) social alienation].*

*H5: Subjective career success will significantly mediate the relationship between social support and pre-retirement anxiety [(a) financial preparedness, (b) social obligation, and (c) social alienation].*

**Figure 1:**
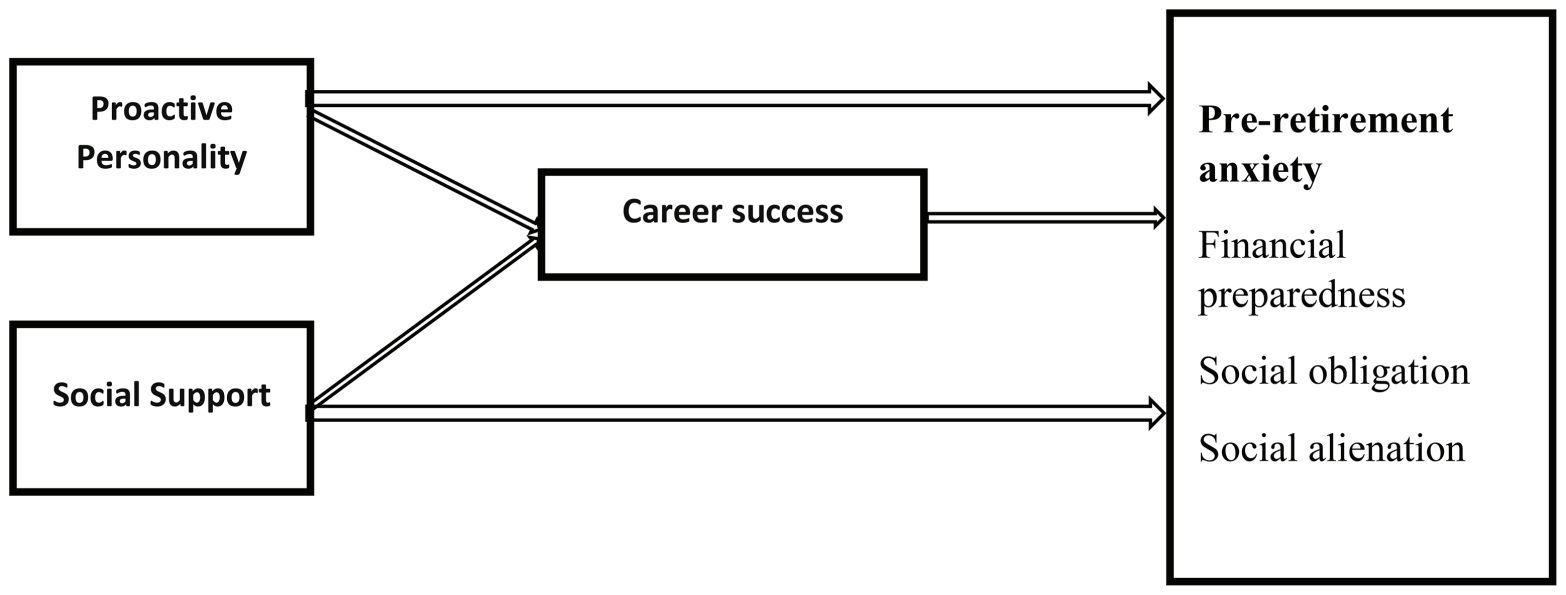
Conceptual model of the mediating roles of career between proactive personality, social support, and pre-retirement anxiety.

Proactive people do not wait for life to happen to them, they take the initiative to shape the world around them. They keep up with growing changes and remain relevant in the streams of things around them. In order to remain relevant, individuals need to intentionally keep up with trends and regularly update their knowledge and skills. This behavior could be the furtherance of education and training as well as investments. This intentional involvement in self-development has to do with taking initiatives and risks needs for positive growth. This perspective is elaborated in the life span development principle of the life course theory. The development does not ends at 18 years, meaning that as individuals experience changes in their lives (physiologically and psychologically) nor ends at work retirement. The skills and knowledge they acquired over the years could well be relied upon after retirement (Figure 1).

As they strive to maintain steady growth, they need not forget that those around them also share in their success story and the best fans they can always have in their lives. The responsibility to the family, friends, and others who depend on them will contribute to their success. The principle of linked lives helps people to make choices, belong to families, churches, etc. that would ease the challenges of life.

This growth comes with financial benefits, status, and recognition. The focus on success and the rewards that comes with it is assumed to be shared among the social network of the individual. The detachment from the social network of the individual as a result of the pursuit of success becomes detrimental as one approaches retirement. The rewards would be around, but the satisfaction that accompanies it will be short-lived as a result of loneliness, depression, and anxiety of the unknown. The choices that people make at work are influenced by what they value, pay, status, service to others, etc., in line with the principle of agency.

## Materials and Methods

### Participants

Participants in this study comprised 624 civil servants from tertiary institutions in the state capitals of six states. Using simple cluster sampling, a state was picked using a table of random numbers from the SPSS v17 (SPSS Inc., Chicago, IL, United States) from each of the six geopolitical zones of Nigeria. Participants who have 5 years or less to retirement were the target population because retirement thoughts could be of concern within this period (Ode, 2004; Maurer and Chapman, 2017). Of the total sample, 237 (37.98%) were males and 387 (62.02%) were females. Their mean age was 56.49 (*SD* = 4.56), ranging from 55 to 66 years. Majority of the participants were married 556 (89.10%), 18 (2.89%) were single, and 50 (8.01%) were widows. The educational level of the participants is as follows: First School Leaving Certificate holders (*n* = 12, 1.92%), Senior School Certificate holders (*n* = 17, 2.72%), Ordinary Diploma (*n* = 167, 26.76%), First degree holders (*n* = 192, 30.77%), Master’s degree holders (*n* = 130, 20.83%), and Ph.D. holders (*n* = 106, 17.05%).

### Measures

#### Proactive Personality Scale

Proactive personality was measured by the Proactive Personality Scale developed by Crant and Bateman (2000). The 10-item proactive personality scale assessed the degree of individual efforts in manipulating the difficult work environment by creating and taking opportunities to gain positive outcomes. Sample items include, “I am constantly on the lookout for new ways to improve my life” and “No matter what the odds, I believe in something I will make it happen.” Each of the questions asked how strongly the respondents agreed or disagreed with proactive personality statements on a five-point scale that ranged from 1 = strongly disagree to 5 = strongly agree. High scores indicate a higher proactive personality and lower scores indicate a low proactive personality. Crant and Bateman (2000) obtained a reliability index (Cronbach’s alpha) of 0.85.

#### Multidimensional Scale of Perceived Social Support

The Multidimensional Scale of Perceived Social Support was developed by Zimet et al. (1988) to measure perceptions and satisfaction with social support. It is a 12-item scale with a seven-point response format ranging from 1 = very strongly disagree to 7 = very strongly agree. The scale has three dimensions: items 1, 2, 5, and 10 measuring support from significant other, for example, “there is a special person who is around when I am in need” and “there is a special person with whom I can share my joys and sorrows”; items 3, 4, 8, and 11 measuring support from family, for example, “I can talk about my problems with my family”; items 6, 7, 9, and 12 measuring support from friends, for example, “My friends really try to help me.” Zimet et al. (1990) reported α coefficients of 0.88 for the total scale, 0.87 for the family subscale, 0.85 for the friend subscale, and 0.91 for the significant others subscale.

#### Subjective Career Success Scale

The Subjective Career Success Scale is a measure for career success developed by Greenhaus et al. (1990). The five-item subjective career success scale measures the perception of employees and their satisfaction with reference to personal financial/nonfinancial goals and achievements. Sample items include, “I am satisfied with the success I have achieved in my career” and “I am satisfied with the progress I have made toward meeting my goals for income.” Items are rated on a five-point scale ranging from 1 = strongly disagree to 5 = strongly agree. Greenhaus et al. (1990) reported a reliability index of the Cronbach alpha of 0.89.

#### Nigerian Pre-retirement Anxiety Scale

The 15-item Nigerian Pre-retirement Anxiety Scale (NPAS) developed by Ugwu et al. (2019) was used to assess the pre-retirement anxiety of potential retirees. Responses are patterned on a five-point scale ranging from 1 = strongly disagree to 5 = strongly agree. Respondents are expected to indicate the extent to which they agree with the items regarding their present level of pre-retirement anxiety. Items 8, 9, 10, 11, and 14 were reversed scored. After reverse-scoring the negatively worded items, higher scores indicate higher pre-retirement anxiety and lower scores indicate lower pre-retirement anxiety levels. The NPAS has three dimensions, which are five-item financial preparedness that deals with financial savings and investments, for example, “I have some reliable source of income after retirement to meet my lifestyle”; six-item social obligation, which deals with basic duties expected from an individual as a result of his/her status for example, “I feel I have not provided for my basic necessities before my retirement (housing, car, etc.)”; and four-item social alienation, which deals with the fear of being neglected and irrelevance in the family or society, for example, “I am afraid I will be lonely when I retire.” Ugwu et al. (2019) reported a reliability coefficient of 0.72, 0.71, and 0.75 for the three dimensions, respectively.

### Procedure

We recruited and trained six research assistants who distributed the questionnaire in each of the states. The research assistants were civil servants, lecturers, and students who are familiar with procedures of the field data collection. They helped in the distribution and collection of the questionnaire for a period of the 2-month interval. All participants were informed that their participation was voluntary, and that data would remain confidential. About 1,200 copies of the questionnaire were distributed (200 in each of the six states surveyed) with the help of the research assistants. The target population was filtered out from the collected questionnaires. Each set of the questionnaire could be completed in approximately 10 min. After completion and collection, properly filled copies of the questionnaire were used for the analysis. Given that our goal was geared toward covering Nigeria as a nation, one state from each of the six geopolitical zones of Nigeria was sampled. The rationale for including participants with 5 years to retirement and excluding those with more than 5 years is based on the fact that people whose retirement looms near tend to naturally feel more concerned about their retirement (Barnes-Farrell, 2003).

A total of 937 copies of the questionnaire were returned, whereas 624 valid copies were used for the analysis after filtering based on the age limit required (5 years to retirement), yielding a valid response rate of 52% out of 1,200 copies of the questionnaire that were initially distributed.

### Design and Statistics

The study adopted a two-wave longitudinal design with a time lag of 2 months apart. Participants were employees from the civil service (staff of tertiary institutions across the country). Purposively selected 1,200 employees were provided with printed questionnaires. Data were gathered in two waves, with 2-month intervals. The reason for collecting data in two waves was to achieve the minimum waves necessary to test the mediation model (Cole and Maxwell, 2003). In order to match the responses of all two waves, each individual was asked to use an initial for easy identification. All waves of survey obtained self-ratings of employees about relational coordination, job satisfaction, and the use of high-performance work practices. Ratings of all study variables were obtained in two waves. However, the data were used according to the need for analytical procedures. The control variables (gender, age, marital status, number of children, and financial dependents) were surveyed only in the first wave. After looking for missing values, 912 responses were usable from first-wave survey (76%). In the second-wave survey, only those 653 employees were willing to continue the study. Thus, the second-wave questionnaires were distributed among 653 employees. After looking for missing values and matching the first- and second-wave responses, only 624 responses (52%) were usable. The response rate from the initial sample to the final usable data is 52%.

Our analysis involves four steps (Clair et al., 2015). In the first step, we measured the reliability and validity of the measurement model using the SmartPLS v3.2.8 (SmartPLS GmbH, Bönningstedt, Germany; Ringle et al., 2015) alongside the fit indices using the IBM SPSS Amos v24 (IBM Corp., Armonk, NY, United States; Arbuckle, 2016). These procedures enable us to validate the psychometric properties of our measures of individual constructs before proceeding to the testing and interpreting structural relations among the constructs. The second step involves the Pearson correlation (*r*) analysis conducted among the study predictors and criterion variables using the IBM SPSS v24 (IBM Corp., Armonk, NY, United States). According to Cohen (1988, 1992), the effect size is low if the value of *r* varies around 0.1, medium if *r* varies around 0.3, and large if *r* varies more than 0.5. The third step was a *post hoc t*-test on the control variable, gender, to confirm the influence of the control variable (gender) on the outcome variable. This is important because previous studies have noted the influence of gender on retirement (Obsorne, 2017; Niles et al., 2018). However, the fourth step comprises testing the direction of the independent variables on the dependent variable and the mediating role of subjective career success applying the path analysis using the IBM SPSS Amos v24 (IBM Corp., Armonk, NY, United States; Arbuckle, 2016) in order to evaluate the structural model.

## Results

To test for the measurement model, we used the SmartPLS v3.2.8 (SmartPLS GmbH, Bönningstedt, Germany) to obtain factor loadings, composite reliability, Cronbach’s alpha, and average variance extracted (AVE). The value of AVE should be greater or equal to 0.5 in order to achieve this validity (Hair et al., 2018; Table 1). The construct validity is achieved when the fitness indices achieve the level of Goodness of Fit Index (GFI), Comparative Fit Index (CFI), and root mean square error of approximation (RMSEA), and standardized root mean square residual (SRMR). The quality of a test is also gauged on the basis of the reliability (internal reliability and composite reliability) of scores from it (Oppenheim, 1992).

**Table 1:** Measurement model.

	Factor loading		Composite reliability	rho_A	Cronbach’s alpha	AVE
Financial preparedness	FP10	0.547	0.647	0.755	0.729	0.540
	FP11	0.758				
	FP14	0.119				
	FP8	0.687				
	FP9	0.584				
Social obligation	SO2	0.568	0.784	0.966	0.828	0.631
	SO3	0.395				
	SO4	0.462				
	SO5	0.991				
	SO6	0.634				
	SO1	0.273				
Social alienation	SA12	0.923	0.477	0.781	0.711	0.567
	SA13	0.324				
	SA15	0.330				
	SA7	0.059				
Proactive personality	PP1	0.695	0.881	0.826	0.871	0.488
	PP10	0.643				
	PP2	0.641				
	PP3	0.466				
	PP4	0.698				
	PP5	0.662				
	PP6	0.643				
	PP7	0.724				
	PP8	0.645				
	PP9	0.718				
Social support (family)	SP1	0.757	0.904	0.914	0.903	0.705
	SP10	0.958				
	SP2	0.763				
	SP5	0.865				
Social support (friends)	SP12	0.746	0.916	0.936	0.915	0.734
	SP6	0.976				
	SP7	0.800				
	SP9	0.887				
Social support (others)	SP11	0.771	0.864	0.865	0.864	0.614
	SP3	0.778				
	SP4	0.826				
	SP8	0.757				
Subjective career success	CS1	0.741	0.908	0.910	0.908	0.664
	CS2	0.800				
	CS3	0.820				
	CS4	0.851				
	CS5	0.859				

AVE, average variance extracted.

In addition, the fit indices of the measurement model, *χ*^2^ = 40.083, *χ*^2^/*df* = 2.358, GFI = 0.97, CFI = 0.97, RMSEA = 0.079, and SRMR = 0.079 all showed that the model provided a good fit to the data (Hu and Bentler, 1999; Hair et al., 2010). We also compared the extent of fit to the data of the hypothesized eight-factor model (i.e., proactive personality; social support: friends, family, and others; subjective career success; and pre-retirement anxiety: financial preparedness, social obligation, and social alienation) with that of an alternative six-factor model (i.e., proactive personality; social support; subjective career success; and pre-retirement anxiety: financial preparedness, social obligation, and social alienation) using the following fit indices: Akaike’s information criterion (AIC), Bayesian information criterion (BIC), consistent Akaike’s information criterion (CAIC), and expected cross-validation index (ECVI). In these fit indices, the lower the values the better the model. For the alternative six-factor model, AIC = 162.083, BIC = 368.537, CAIC = 429.537, and ECVI = 0.747, whereas for the hypothesized eight-factor model, AIC = 119.068, BIC = 240.910, CAIC = 276.910, and ECVI = 0.549. Based on the results, the hypothesized model displayed a better fit with the data relative to the alternative model. Thus, the hypothesized model was selected.

The Pearson’s correlation was performed to ascertain the associations of the study variables including the demographic variables or control variables (see Table 2). The results revealed that there was a significant relationship between gender (dummy coded “0” = female and “1” = male) and financial preparedness and social obligation (*r* = 0.44, *p* = 0.001 and *r* = 0.14, *p* = 0.037, respectively), indicating that males were more associated with anxiety relating to financial preparedness and social obligation than females. Age was significantly and positively related to social alienation (*r* = 0.28, *p* = 0.007) but was not significantly correlated with social obligation and financial preparedness. This reveals that the older they are, the more likely socially alienated they felt. Multiple-level dummy category was created for marital status (single, married, separated/divorced, and widowed), it was found that singles were associated with financial preparedness and social alienation anxiety (*r* = 0.21, *p* = 0.002 and *r* = 0.16, *p* = 0.017, respectively), whereas married were found to be less likely to be associated with social alienation (*r* = −0.13, *p* = 0.015), and separated/divorced and widowed were equally found to be associated with social alienation (*r* = 0.17, *p* = 0.016 and *r* = 0.22, *p* = 0.005, respectively). Number of children had a significant positive relationship with social obligation (*r* = 0.38, *p* = 0.001) but not financial preparedness and social alienation. Financially dependent had a significant positive relationship with social obligation (*r* = 0.28, *p* = 0.001) but not financial preparedness and social alienation. Proactive personality had a negative relationship with financial preparedness (*r* = −58, *p* = 0.001), social obligation (*r* = −0.32, *p* = 0.001), and social alienation (*r* = −31, *p* = 0.001). Career success had a significant negative relationship with financial preparedness (*r* = −0.29, *p* = 0.005), social obligation (*r* = −0.22, *p* = 0.011), and social alienation (*r* = −0.11, *p* = 0.043). Social support (family) had a significant negative relationship with financial preparedness (*r* = −0.16, *p* = 0.041), social alienation (*r* = −0.14, *p* = 0.048), and social obligation (*r* = −0.16, *p* = 0.032). Social support (friend) had a significant negative relationship with financial preparedness (*r* = 0.45, *p* = 0.001) but not with social obligation and social alienation. Social support (other) had a significant negative relationship with financial preparedness (*r* = −0.46, *p* = 0.001) but not with social obligation and alienation.

**Table 2:** Descriptive statistics and construct correlation matrix (*N* = 624).

	Variables	1	2	3	4	5	6	7	8	9	10	11	12	13	14	15	16
1	Gender		0.04	−0.11	0.20^∗∗^	−0.20^∗∗^	−0.05	−0.08	−0.06	0.12	0.14^∗^	0.14^∗^	0.08	0.13	0.44^∗∗^	0.14^∗^	0.14
2	Age			−0.43^∗∗^	−0.19^∗∗^	0.30^∗∗^	0.06	0.46^∗∗^	0.33^∗∗^	0.35^∗∗^	0.24^∗∗^	0.26^∗∗^	0.34^∗∗^	0.27^∗∗^	0.05	0.13	0.28^∗∗^
3	Single				0.15^∗^	0.41^∗∗^	0.06	0.17^∗^	0.25^∗∗^	0.24^∗∗^	0.30^∗∗^	0.27^∗∗^	0.32^∗∗^	0.26^∗∗^	0.21^∗∗^	0.05	0.16^∗^
4	Married					−0.59^∗∗^	−0.09	−0.39^∗∗^	−0.29^∗∗^	0.03	0.18^∗∗^	0.00	0.09	0.04	−0.01	−0.02	−0.13^∗^
5	Separated/divorced						−0.25^∗∗^	0.41^∗∗^	0.44^∗∗^	0.14^∗^	−0.09	0.14^∗^	0.08	0.07	0.05	0.02	0.17^∗^
6	Widowed							−0.03	−0.08	−0.11	0.12	0.04	−0.03	0.02	0.10	0.05	0.22^∗∗^
7	Number of children								0.69^∗∗^	−0.01	0.03	0.06	0.08	0.06	0.05	0.38^∗∗^	−0.10
8	Financially dependents									0.050	−0.04	0.03	0.13	0.06	0.00	0.28^∗∗^	0.06
9	Proactive personality										−0.38^∗∗^	−0.47^∗∗^	−0.33^∗∗^	−0.47^∗∗^	−0.58^∗∗^	−0.32^∗∗^	−0.31^∗∗^
10	Career success											−0.48^∗∗^	−0.46^∗∗^	−0.50^∗∗^	−0.29^∗∗^	−0.22^∗∗^	−0.11^∗∗^
11	SS family												−0.70^∗∗^	−0.81^∗∗^	−0.16^∗^	−0.14^∗^	−0.16^∗^
12	SS friends													−0.77^∗∗^	−0.45^∗∗^	0.01	0.08
13	SS others														−0.46^∗∗^	0.02	0.07
14	Financial preparedness															−0.07	−0.26^∗∗^
15	Social obligation																−0.41^∗∗^
16	Social alienation																
	M		56.49					3.68	4.41	37.24	15.01	19.48	17.40	19.80	12.51	15.91	12.83
	SD		4.58					2.99	2.91	8.72	6.04	6.98	7.02	6.57	3.45	5.75	3.81

∗ *p* < 0.05;

∗∗ *p* < 0.01.

Gender was coded as a dummy variable male = 0, female = 1; Marital status: multiple dummy coded. Family SS, family social support; Friend SS, friend social support; other SS, other social support.

### *Post hoc* Test

The *post hoc t*-test result showed that females were less likely to experience higher financial preparedness and social obligation anxiety than males.

### Test of the Structural Model

To test all the hypotheses and the overall mediation model shown above, the mediation analysis was conducted using the IBM SPSS Amos v24 (IBM Corp., Armonk, NY, United States) controlling for age, gender, marital status, number of children, and financial dependents. Finally, a bootstrap was conducted to ensure that the mediation indirect effect was actually significant and reduce error variance, bias, and prediction error.

**Figure 2:**
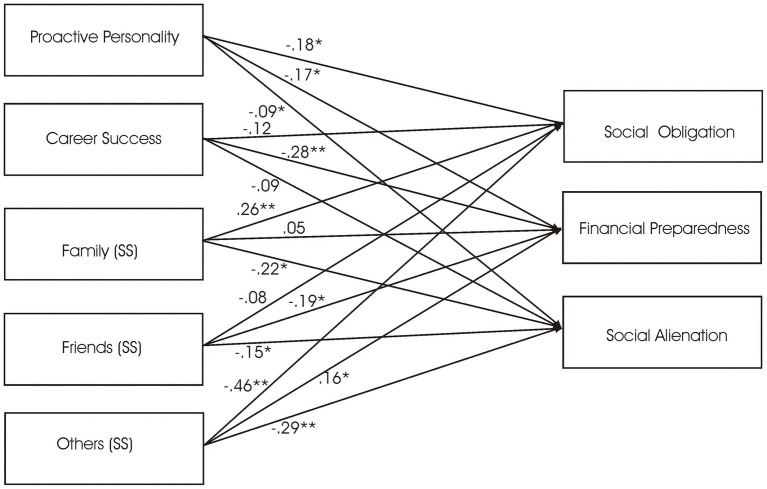
Regression results of relationships of proactive personality, career success, social support (family, friends, and others), and pre-retirement anxiety. ^∗^*p* < 0.05 and ^∗∗^*p* < 0.01 Covariates = gender, age, marital status, number of children, and financial dependents.

It was shown in Figure 2 that proactive personality was significantly associated with social obligation, financial preparedness, and social alienation (*β* = −0.18, *p* = 0.037; *β* = −0.17, *p* = 0.045; and *β* = −0.09, *p* = 0.048, respectively, supporting H_1_). Social support (family) was significantly associated with social obligation and social alienation (*β* = 0.26, *p* = 0.044 and *β* = −0.22, *p* = 0.048, respectively). Social support (friends) was significantly associated to financial preparedness and social alienation (*β* = −0.19, *p* < 0.05 and *β* = −0.15, *p* < 0.05), whereas social support (others) was significantly associated with social obligation, financial preparedness, and social alienation (*β* = 0.46, *p* < 0.001; *β* = 0.16, *p* > 0.05; and *β* = 0.29, *p* < 0.05, respectively, supporting H_2_). Subjective career success was significantly associated with financial preparedness (*β* = −0.28, *p* < 0.001) but not social obligation and social alienation (partly supporting H_3_).

Subjective career success mediated the relationship between proactive personality and pre-retirement anxiety (social obligation, financial preparedness, and social alienation). This shows that subjective career success had a mediating effect on the relationship between proactive personality and pre-retirement anxiety (social obligation, financial preparedness, and social alienation; supporting H_4_). In the first set of mediation analysis that showed subjective career success did not mediate the relationship between social support (others) and pre-retirement anxiety, whereas the mediation analysis shows that subjective career success mediated the relationship between social support (friends) and pre-retirement anxiety (social obligation and financial preparedness, supporting partly H_5_). We used bootstrap confidence intervals as a test of significance to test the mediational model of subjective career success as a mediator of the relationship between proactive personality, social support, and pre-retirement anxiety at different levels (Table 3).

**Table 3:** Path analysis test decomposition of effects (*n* = 624).

Variables	Coefficient	SE	BC 95% CI		*p*
			LL	UL	
Proactive personality → (SCS) →social obligation	−0.035	0.017	0.054	0.010	0.000
Proactive personality → (SCS) →financial preparedness	−0.033	0.023	0.012	0.109	0.004
Proactive personality → (SCS) →social alienation	−0.023	0.018	0.015	0.049	0.014
SS friends → (SCS) →social obligation	−0.021	0.029	0.059	0.088	0.023
SS friends → (SCS) →financial preparedness	0.044	0.015	−0.008	0.107	0.086
SS friends → (SCS) →social alienation	0.011	0.017	−0.011	0.064	0.287
SS family → (SCS) →social obligation	−0.009	0.034	−0.066	0.007	0.269
SS family → (SCS) →financial preparedness	0.037	0.016	−0.017	0.119	0.184
SS family → (SCS) →social alienation	0.009	0.016	−0.009	0.064	0.280
SS Others → (SCS) →social obligation	−0.011	0.018	−0.080	0.007	0.214
SS Others → (SCS) →financial preparedness	0.045	0.038	−0.011	0.146	0.117
SS Others → (SCS) →social alienation	0.011	0.019	−0.010	0.076	0.246

Bootstrapping results were based on 5,000 bootstrapped samples. SE, standard error; BC, bias corrected; CI, confidence interval, LL, lower limit; UL, upper limit; SS, social support; SCS, subjective career success.

## Discussion

The main objective of this study was to determine whether subjective career success would mediate the relationship between proactive personality, social support, and pre-retirement anxiety. The first hypothesis of the study predicted a significant negative relationship between proactive personality and pre-retirement anxiety (financial preparedness, social obligation, and social alienation).

The result of the path analysis showed that proactive personality was a significant negative predictor of social obligation but was not a significant predictor of the other two dimensions of pre-retirement anxiety (financial preparedness and social alienation). The hypothesis was therefore not rejected. The significant relationship between proactive personality and social obligation is supported by some studies (Lockenhoff et al., 2009; Robinson et al., 2010; Claes and Van Loo, 2011; Feldman and Beehr, 2011; Jawahar et al., 2012), which point to the fact that those who adapt or make necessary adjustments to fit into the changing world and work demands were more likely to remain relevant and contribute to their work and life after retirement than those who are passive.

Social obligation, which is the feeling that people have about fulfilling their social obligation to themselves, family, and society, can induce anxiety when they fail at it. Some of these obligations could be like taking care of their health, welfare of their family (housing, education, and psychological and economic support), and assisting friends and relatives when they needed help. From the findings, the failure could be as a result of their not taking initiative, taking charge and taking the basic risks required of them at the earlier stages of their work–life to ensure that they fulfill these obligations. The proactive behavior could be taking up new challenges in improving themselves for a better job and supporting their families even with the little they have. The nonsignificant relationship between proactive personality and financial preparedness was in contrast to some studies (e.g., Wawoe, 2010; Claes and Van Loo, 2011), which submitted that proactive people have higher tendencies to save or plan for future earnings than less proactive people. The basic tendencies of proactive people make it easy for them to have a bigger picture of the world around them than the mere moment. This result could trigger a sense of helplessness in controlling their income. The policies and constant changes in political decisions of leaders could affect their plans financially by delayed payment of salaries, unpredictable economic trends, etc.

From the finding of this study, proactive personality was not a significant predictor of financial preparedness. This was in contrast to the study of Hershey and Mowen (2000) and Segel-Karpas and Werner (2014), who posited that proactivity influences pre-retirement financial planning behavior. The difference in the findings could be as a result of the organizational type (private and public sector) that the participants belong to, which induces risk aversive tendencies in its works (Idowu and Olanike, 2010). The organizational type of which the previous studies were conducted was not specified, but it was observed that they tested for financial knowledge. Public organization workers (public sector) are probably comfortable with the job security and pension safety net they feel will cover their retirement demands and possibly do not seek financial knowledge within or outside the organization. However, most workers with private organizations are constantly on the lookout for means to secure themselves considering the fact that they could lose their job anytime. It can be assumed that the participants in the study were exposed to financial knowledge, which influenced their decisions to make early plans on savings and starting a business to keep them liquidated after retirement (Denton et al., 2004; McCullough, 2012).

Proactive personality was also found not to predict social alienation; this was also in contrast to the study of Adams et al. (2004), who posited that as people approach retirement, they get depressed and feel lonely. This study showed that proactivity does not directly lead to social alienation. The relationship between proactive personality and social alienation could be indirect as there is a possibility that the relationship could be complex or nonlinear, meaning that it could be moderated or mediated by another factor.

The second hypothesis of the study predicted a significant negative relationship between social support (friends, family, and significant other) and pre-retirement anxiety (financial preparedness, social obligation, and social alienation). The results of the path analysis clearly showed that family social support was a significant negative predictor of social obligation. This means that the higher support an individual perceives he/she receives or hopes to receive from his/her family network, the lower the chances that he/she is going to experience anxiety pertaining to social obligation before retirement. This is consistent with some findings (e.g., van Tilburg, 1992; Price, 1998), which argued that as people approach retirement, the family relationship is considerably reflected as this could affect the well-being of the retiree after retirement. This also can be true as the individual retires, there exists a disconnect between his/her family members from his/her coworkers. The closest people around the person are his/her family, both immediate and extended.

The result further showed that there was a significant negative relationship between social support from significant other and social obligation but not financial preparedness and social alienation. This also shows that the higher one feels supported by his/her significant other, the lower the anxiety associated with approaching retirement. This finding is also consistent with the outcome of some studies (e.g., Casale et al., 2015; Kugbey et al., 2015; Rapee et al., 2015; Roohafza et al., 2015; Lilympaki et al., 2016). The results posit that people care about their relationships with others outside their families. These significant others could be coworkers, neighbors, and the relationship with religious organizations or societies they belonged to. These significant others could be of immense help to them upon retirement in diverse ways. The relationship established with coworkers, neighbors, or religious affiliations makes it easy for an individual to ask for favors for self or family and gain access to some resources, which normally would have required longer processes. For example, a coworker who is a medical doctor can be reached later for free consultancy. Furthermore, the information about employment/contracts/grants opportunities can be gotten for self or family members as a result of affiliation to a significant other.

Although the social support of friends was not significant with any of the dimensions of pre-retirement anxiety, the finding is in contrast with previous studies (e.g., Takagishi et al., 2011; Casale et al., 2015; Kugbey et al., 2015; Rapee et al., 2015; Roohafza et al., 2015; Lilympaki et al., 2016), which found that friends play a significant role in reducing anxiety in various incidences of lives of people like retirement. But it can be argued that confusion of the concept of friendship and others outside the family could be responsible as it is possible that anybody close to the individual is seen as a family member, whereas those not too close are considered outsiders and can also be construed as friends.

The third hypothesis of the study predicted a significant negative relationship between career success and pre-retirement anxiety (financial preparedness, social obligation, and social alienation). Career success was only a significant negative predictor of financial preparedness but not social obligation and social alienation. The third hypothesis was not rejected (financial preparedness). This could possibly be that as people gain satisfaction with their careers, their fear of financial wants dissipates as they never chose the career because of financial gains, and it could also be as a result of the experienced returns and expected return on social capital investments they must have made in lives of many people throughout the course of their career. This shows that as people aspire and attain higher career satisfaction, the effects of financial challenges are minimized. Higher career success has been associated with higher financial satisfaction. This is in line with previous studies (e.g., Quadagno, 1978; Brooke, 2009; van Dam et al., 2009; Damman et al., 2013; De Vos and Segers, 2013; De Coen et al., 2015). From the studies reviewed, it was observed that some reasons why some people experience anxiety are because the benefits expected from their career are not coming or that the benefits they are currently enjoying might come to an end soon after retirement. Another possible reason could be the failure to realize that age and trends were changing (meaning that as time passes and knowledge grows, one is expected to upgrade to flow with trends; thus, it presents opportunities for promotion and consequently higher financial benefits; van Dam et al., 2009) around their works, for example, teachers (Arogundade, 2016), physicians (Quadagno, 1978), and IT professionals (Brooke, 2009), who failed to plan. They desire to delay their retirement, whereas some others were forced to retire unprepared as a result of poor performance and poor health (Damman et al., 2013). All these show challenges toward adequate financial preparedness. The unpredictability of trends (economically and politically) in the work ecosystem of Nigeria can present challenges to workers to prepare financially for retirement as some of these unpredictable factors can make a worker spend his/her savings before retirement, leaving them relatively empty at retirement. The hypothesis was therefore confirmed.

The fourth hypothesis, which states that career success will significantly mediate the relationship between proactive personality and pre-retirement anxiety (financial preparedness, social obligation, and social alienation) among Nigerian civil servants, was significant. The fourth hypothesis was also not rejected. This finding is in line with the results of previous studies (e.g., Lockenhoff et al., 2009; Sutin et al., 2009; Sutin and Costa, 2010; Feldman and Beehr, 2011), which showed that proactive workers learn quickly to adjust or adapt to changing trends in their abilities and work environment to fit into the requirements of their work. Career success has shown to mitigate the anxiety that comes with retirement as cited by previous studies (e.g., Armstrong-Stassen and Cattaneo, 2010; De Coen et al., 2015), which posited that those high on career success tend to be more effective and enjoy their work–life so they desire to continue. Thus, they achieve in a less demanding position. With great satisfaction with their work, the idea of retirement is nonexistent (e.g., Wingrove and Slevin, 1991; Armstrong-Stassen and Cattaneo, 2010; De Vos and Segers, 2013; De Coen et al., 2015). These tendencies have been found among proactive individuals who make deliberate choices in changing their work and world and follow their desired career path. This can be seen among some retired academicians and military personnel who end up working with research institutes and non-governmental organizations or engage in their own personal projects. The hypothesis was therefore confirmed. The results of this study lead one to consider the role that personality dimension could play in the pursuits of happiness and life satisfaction of a worker as opposed to relying solely on the social status and rank of a worker and the reputation of the organization or institution. The proactive individual is one who identifies opportunities and does not shy away from unfamiliar situations. The proactive individual generates change constructively to enhance his/her experience and outcomes (Maurer and Chapman, 2017). Proactive personality from this study can be opined to be positively related to satisfaction with work that is the opposite of pre-retirement anxiety, which indicates that having a proactive personality is associated with a more positive experience as one moves away from work and becomes fully retired. Perhaps the end of career and the beginning of retirement is not perceived as a stressful disruption or unwelcomed event for the proactive person, but instead, retirement represents a new context and a new challenge, which is surmountable and which will be managed to his/her benefits.

The fifth hypothesis, which states that career success will significantly mediate the relationship between social support (family, friends, and others) and pre-retirement anxiety (financial preparedness, social obligation, and alienation) among Nigerian civil servants, was found to be significant among social support (friends) and pre-retirement anxiety (financial preparedness). This can be explained as a situation where those with quality friendship and high career success experience less anxiety with their financial preparedness. In their study, Taylor et al. (2008) examined the effects of social support on post-retirement adjustment *via* a longitudinal study. Results suggested that social support consistently and significantly predicted satisfaction early and later in retirement. Moreover, Obodo (2017) investigated social support as a predictor of pre-retirement anxiety among prospective retirees in the civil service within Enugu metropolis. Results revealed that social support negatively predicted pre-retirement anxiety, implying that prospective retirees with poor social support/network have high levels of anxiety toward retirement. Furthermore, Amorim et al. (2017) examined predictors of happiness among retirees from urban and rural areas in Brazil. Results identified healthy social support and economic situation as important predictors of happiness, but no moderation effects of urban and rural areas were found. Based on the review of social support literature on retirement satisfaction, it could be said that social support is a potent mediator in the relationship between retirement satisfaction and its antecedents. This implies that the presence of social support is likely to make up for limitations in leisure satisfaction, health quality, and career success among retiring employees (Umukoro and Adejuwon, 2017). These findings are supported by research on human relationships, such as the MacArthur studies of aging (Rowe and Kahn, 1998), which validated the link of social relationships to loneliness and depression. Those who have a great deal of social support, such as quality relationships, financial resources, and a network of supportive family members, tend to be healthier than individuals who lack such support (Bosworth and Schaie, 1997).

## Implications of the Findings

The research findings revealed the roles that subjective career success plays in the relationship between proactive personality, social support, and pre-retirement anxiety. The dimensions studied show that retirement challenges are not just on financial inadequacies, but the challenges of not meeting some social obligations and the fear of being alienated. For the individual, the findings draw attention to some social and personality characteristics that need to be considered all through work–life of individuals, such as family, friends, and pursuit of job satisfaction.

The findings point to the need for an early intervention for pre-retirees in various organizations to build resources, socially and emotionally. This could be achieved by adopting the resource-oriented group intervention technique on retirement by Seiferling and Michel (2017) who have developed cognitive techniques to help pre-retirees to prepare for their transition to retirement by breaking cognitive barriers, limiting intentional future plans. Specifically, these techniques could be reproduced across the organizations and incorporated into the training and development programs for employees. This way, as soon as employees get into service, they come in contact with structured orientation programs on how to prepare for retirement. By the pre-retirement stage, therefore, they are expected to be better positioned for transitioning.

For governments, these findings point to the need for structured policies that address the retirement process. For instance, it is important that the government begins to formulate policies that favor pre-retirees within the organizations and which address the need for employers to focus attention on early intervention for pre-retirees.

Furthermore, the findings from this study have implications for directing future research in understanding retirement adjustment, a field that is rapidly growing in terms of interest and need. The literature has not yet adequately covered the specific relation between proactive personality, social support, and retirement adjustment, and this relation may have a substantial impact on how we understand this adjustment process. In the domain of practice, there is a potential for intervention tailored for people with different levels of personality traits in order to increase their chances of experiencing a positive transition to retirement.

## Limitations of the Study and Future Research

Although the methodology deals adequately with the manifest variables, critical latent variables cannot be suitably accounted for by the use of purely quantitative instruments. Due to the complex and multifaceted nature of pre-retirement, a deeper understanding of the roles of proactive personality, social support subjective career success, and pre-retirement can be attained using both quantitative and qualitative approaches. Perhaps qualitative approaches, such as interviews with pre-retirees, pension scheme stakeholders, and employers, can provide insights and answers to some questions in this field of scientific inquiry. This mixed-method approach in investigating pre-retirement is worthwhile and should be considered by future researchers.

It is remarkable that research has linked proactive personality, social support, and subjective career success to feelings, such as pre-retirement anxiety, but retirement planning and decision-making were not addressed on how proactive personality and social support will influence it (Topa et al., 2009). The present study suggests that research might explore whether proactive personality is a predictor of retirement planning and preparation, and the preparation contributes to life satisfaction in retirement.

## Conclusion

This study found the immense role of social support systems in the lives of individuals before retirement, especially in Africans who take pride in family and friends. Moreover, this study found that there are some complex relationships that could induce anxiety among pre-retirees, such as the indirect role of career success in facilitating the influence of proactivity and social support on pre-retirement anxiety.

Finally, pre-retirement anxiety among workers approaching retirement can be said to be enhanced or alleviated by understanding the complex web of connections with factors, such as proactivity, social support, and subjective career success.

## Data Availability Statement

The raw data supporting the conclusions of this article will be made available by the authors, without undue reservation.

## Ethics Statement

The studies involving human participants were reviewed and approved by Dr. Chijioke Odii Renaissance University Enugu. The patients/participants provided their written informed consent to participate in this study.

## Author Contributions

LU conceived the idea, developed the initial draft, and collected the data and analyzed it. IE developed the draft. BN developed the theoretical framework. LU and IE interpreted the findings and discussed the results while SU, FO, EN, AE, CE, and ME read and editing the final manuscript. All authors contributed to the article and approved the submitted version.

### Conflict of Interest

The authors declare that the research was conducted in the absence of any commercial or financial relationships that could be construed as a potential conflict of interest.
